# Association between Internet use and adolescent behavioral development: a cross-lagged regression study

**DOI:** 10.3389/fnbeh.2025.1654194

**Published:** 2026-02-05

**Authors:** Desheng Yan, Lei Qi, Yao Chen, Guangming Li

**Affiliations:** 1 Inner Mongolia Minzu Preschool Education College, Ordos, China; 2 Baotou Teachers’ College, Inner Mongolia University of Science and Technology, Baotou, China; 3 Longsai Senior High School, Ningbo, China; 4 School of Psychology, South China Normal University, Guangzhou, China

**Keywords:** adolescent behavioral development, cross-lagged regression, Internet use behavior, prohibitive behavior, promotive behavior

## Abstract

Promotive behavior refers to actions that facilitate individuals’ efforts to surmount obstacles and proactively pursue their goals, thereby fostering adaptive functioning and positive development. In contrast, prohibitive behavior refers to harmful or otherwise maladaptive actions that may hinder individuals’ personal growth and developmental outcomes. This study aims to explore the possible causal relationship between Internet use behavior and adolescent behavioral development, including promotive behavior and prohibitive behavior. There were 9,132 students’ data that were analyzed, and cross-lagged regression analysis was used to study causal relationships between two or more variables that change over time. Data were collected at two time points: T1 (2013–2014) and T2 (2014–2015), with an interval of approximately 1 year between the two waves. Results indicated a negative correlation between Internet use behavior and promotive behavior at points-in-time T1 and T2, while a positive relationship was observed between Internet use behavior and prohibitive behavior at both time points. Findings suggest that reducing problematic Internet use among adolescents contributes to the development of healthy behaviors, and active and healthy adolescent behaviors can in turn reduce their problematic Internet use.

## 1 Introduction

Adolescence is a crucial time for developing positive behaviors and setting the groundwork for a happy life in the future. Therefore, the development of teenage behavior has always been the subject of educational psychology study, the primary responsibility of educators, and a prerequisite for instruction. The idea of teenage conduct has a long history. It has mostly been studied in psychology, with an emphasis on externalizing behavior as the study focus and adaptive growth and normative behavior. According to some academics, behavioral development occurs on both the inside and the outside, with each fulfilling its purpose and mixing together as bad and positive behaviors alternately emerge and become more like one another. According to behaviorism, an organism’s response and movement to stimulation are what constitute its behavior. Biologists typically refer to behavior as the observable activity of muscles and exocrine glands; philosophers observe that behavior is the external activity that is dominated by thought; and the theory of organizational behavior generalizes behavior as the result and expression of people’s interactions with their surroundings (Yang and Cao, 2002). As a result, behavior is the sum of its subjective and objective components. It is made up of objective exterior variables like the natural environment and the social environment as well as subjective internal aspects like the body, emotions, temperament, character, and values. Because of how these two interact, conduct is both consistent and unpredictable (Yang, 2000).

In summary, most previous studies used survey, cross-sectional, and self-report methods. These approaches depend on the subject’s memory, expression, or introspection and lack objective behavioral standards. Most research also focuses on student groups, which limits sample diversity. The cross-sectional approach cannot provide causal evidence (Liu, 2015). Many studies describe Internet applications and usage, exploring their influences and causes. First, they are broad, covering both Internet + field applications and industry development; Second, they target diverse groups, focusing on network users from various industries; Third, they address focused topics like Internet addiction, cyberbullying, information sharing, and industry trends. However, the effect of Internet use on teenage behavioral development has received less attention. Few studies examine whether teenage behavioral development affects Internet use behavior and how the two interaction dynamically. Most use cross-sectional surveys to analyze just the one-way effect of Internet use behavior on behavior development. In contrast, monitoring data can dynamically and continually record the process behavior and integrate into the interactive mechanism the intricate relationship between adolescent behavioral development and Internet use behavior. Therefore, this study uses a cross-lagged research method to analyze the relationship between Internet use behavior and adolescent behavioral development from a complex and holistic perspective. This approach offers young people useful advice on responsible and productive Internet use in the digital era. In this study, we utilized cross-lagged analysis to explore the relationship between internet use behavior and adolescent behavioral development. This technique enables us to assess the bidirectional effects and changes over time. By using cross-lagged analysis, the researchers aimed to reveal how internet use behavior impacts adolescent behavior development and vice versa at different times.

### 1.1 Promotive behaviors and prohibitive behaviors

According to the results, behavioral reaction can be classified as either excellent or poor. This corresponds to a couple of related concepts: promotive behavior and prohibitive behavior. The term “promotive behavior” describes actions that inspire people to overcome obstacles and work toward their objectives. The Internet has had a profound impact on the formation and expression of adolescents’ promotive and prohibitive behaviors. First, social media platforms provide adolescents with spaces to share experiences and to support their peers, thereby enhancing promotive behaviors in this setting. For example, online support groups and positive interactive feedback can help adolescents overcome obstacles, bolster self-efficacy, and strive toward shared goals. However, Internet anonymity may also contribute to an increase in prohibitive behaviors, such as cyberbullying and malicious remarks, which not only harm victims but may also suppress other adolescents’ positive behaviors. Moreover, information overload is prevalent in online environments and may lead to anxiety and jealousy among adolescents, thereby triggering more prohibitive behaviors. Therefore, understanding how Internet use behavior influences these two types of behaviors among adolescents is of considerable importance for promoting positive online social interactions and reducing negative behaviors.

Previous studies have demonstrated that teenage promotive behavior is essential to their development and enhances their self-efficacy, learning ability, and originality. Prosocial behavior can be viewed as a specific manifestation of promotive behavior, particularly in contexts emphasizing social harmony. Prosocial behavior, for instance, has been linked over time to improved academic success. Fostering prosocial behavior may also assist in reducing aggressive behavior and increasing adolescent study achievement, which in turn is associated with higher test scores and higher levels of self-efficacy (Caprara et al., 2014; Carlo et al., 2018).

Prohibitive behaviors were manifested as swearing, fighting, skipping class, copying homework, staying at Internet bars, game halls, and other similar bad behaviors that restrict the development of adolescents. Machado et al. (2018) provided evidence of a link between Internet addiction and behavioral problems among adolescents. Internet addiction was associated with early adolescents, with heads of households having lower educational attainment, with individuals who spent four or more hours a day surfing the Internet, and with daily Internet use (Bueno et al., 2024). Andrade et al. (2021) surveyed 2,214 students (*M* = 21.9) from across Brazil via an online questionnaire and found that Internet addiction is significantly associated with emotional problems-depression, anxiety, and stress.

Adolescent behavior is highly reversible. Behavior does not exist in isolation; similarly no person is an isolated entity but rather a complete self-reaction to behaviors that align with their cognitive framework and the surrounding situation (Yang and Han, 2003). Existing research showed that due to the external factors of bad environment and peer influences, adolescent behavioral variability is significantly higher than stability, especially in the strong stimulation field where promoting behaviors are susceptible to being influenced, regulated, and transformed into prohibitive behaviors for adolescents, who are more impulsive and emotional (Galván, 2014). It has also been confirmed that the likelihood of initiating addictive behavior is higher during adolescence than at any other stage of development (Gladwin et al., 2011). Existing results also indicate that early adolescent behavior is highly guided by social circumstances across domains. Peer influence can motivate rule-breaking behavior, but it can also promote compliance and develop prosocial behavior (Molleman et al., 2022). Transforming prohibitive into promotive behaviors is crucial for lifelong adolescent development. The influence of the Internet on adolescents is particularly obvious for its unique advantages such as diversity, openness, virtuality, interactivity, and immediacy. In the virtual environment of the network, people are the main body of behavior, which produces different Internet use behaviors (Liu, 2015). The impact of the Internet on teenagers is pronounced. The Internet is an important threshold for the generation of teenagers’ behavior as well as the field of modern human existence. The way of Internet use directly affects the orientation of teenagers’ behavior. In the process of teenagers’ development, it plays an increasingly vital role, but also brings a series of problems while optimizing young people’s studies and lives (Wei et al., 2022).

### 1.2 The dual role of the Internet in adolescent behavior

The Internet can lead to both promotive and prohibitive behaviors. On the one hand, it provides a powerful tool for teenagers to adapt to the development of the times. On the other hand, it is easy to produce prohibitive behavior deviating from the direction of development. Based on this phenomenon, the research about children’s Internet use behavior mainly concentrates on three aspects: network violence, health behavior, and Internet addiction. It focuses on Internet behavior, characteristics, dynamic factors, and cause. Some scholars have pointed out that Internet games can cause physical health problems (Gillespie, 2002), and internet addiction lead to young people’s aggressive behaviors toward others (Ko et al., 2009). Ramos et al. (2025) found that 37.4% of adolescents had engaged in cyber aggression. Those involved exhibited significantly higher levels of problematic smartphone use and problematic Internet use, along with more frequent engagement in risky or harmful online behaviors. When young people suffer from Internet violence in their personal growth experience, they are more likely to engage in other forbidden behaviors (Hellevik, 2019). Li et al. (2015) found that online bullying independently positively predicted deviant behavior, even after controlling for the effects of traditional bullying. Online bullying’s independent explanatory rate was 5.1 per cent of deviant behaviors, while traditional ones were only 2.3 per cent. There are significant differences between Internet addicts and normal Internet users in terms of time spent, place, content, motivation, attitude and evaluation (Zhou et al., 2016). The results of Zhou et al.’s (2014) study indicate the presence of impulsivity, executive function deficits, and working memory impairments among individuals with Internet addiction disorder and those with alcohol dependence. These findings suggest that Internet-addicted individuals exhibit similar patterns of impulsivity and executive dysfunction to those observed in alcohol-dependent individuals.

As an interesting subject with special psychological and physical needs, teenagers cannot avoid the influence brought by the dual attributes of the virtual self-constituted by the Internet in the process of self-development (Gao, 2007). Open cyberspace gives teenagers a free choice opportunity that is full of many uncertainties, which makes it easy to induce them to have a selective bias from facilitating to forbidding behavior. However, the Internet does not allow plenary sessions to be completed, nor does it necessarily lead to the occurrence and intensification of prohibitions. High information literacy enables students to translate Internet-use behaviors into promotive behaviors, reflecting a positive mapping from digital information practices to constructive, goal-directed actions. In the digital era, good digital literacy can improve information processing skills and continuously shape and enhance the self-awareness of behavior. Some scholars expound the development of students’ digital literacy in the “Internet +” era in five aspects of critical thinking, learning to learn, and practical innovation. The learning platform and environment provided by the Internet with the characteristics of sharing, autonomy, interactivity, and collaboration, are increasingly showing the independence of young people’s learning behavior and conscious activity behavior (Song and Tian, 2017).

Internet use behavior leads to the occurrence of corresponding behavior under the intention, and at the same time, Internet use behavior is related to the behavior itself. With the help of the two-way provision perspective of environmental supply, the intelligent environment integrated with technology enriches the interaction behavior of learners and mobilizes their initiative. The coordination model of “human-environment” double-transferring feedback supports that under the complex Internet environment, the intelligent environment can improve its availability through multiple teaching activities so that learners of different states, levels, and types can perceive their learning paths and methods (Wang et al., 2021). This approach makes up for the shortcoming that the bidirectional feedback perspective of environmental behavior perception cannot deeply explore the response and coordination of students’ behavior habits. Hence, the process of interactive behavior between the informational functional environment provided by the Internet and the two-way feedback of the subject is worth studying. The theory of planned behavior holds that behavior choice is determined by attitude. Based on this, Ma (2015) used the structural equation model (SEM) to model and analyze Internet users’ behavior. Combining qualitative and quantitative methods, some scholars arrive at the results that the behavioral attitude of Internet use is positively correlated with users’ behavior, and the former will also have a more direct impact on the latter (Ma, 2015). Attitude and cognition influence behavior, which in turn influences individual performance.

The Internet has changed the way people work and study. Sample data displayed 75% of students believe that their work and study cannot be separated from the help of smartphones and the Internet. Among the influencing factors of working and learning styles, online communication behavior has a significant positive effect (Yu and Liu, 2016). Getting information on the Internet has a prominent positive impact on students’ well-being, while pursuing entertainment on the Internet has the opposite impact on it (Shen et al., 2014). Besides, Internet use has a remarkable influence on different dimensions of students’ lifestyles because different users have different needs for the Internet, which may lead to different behaviors. Correspondingly, the same Internet service function may affect each user differently (Li and You, 2007).

Based on the above analysis, the following study assumption is proposed:

*Hypothesis 1*: Internet use behavior is negatively correlated with promotive behavior among adolescents.

*Hypothesis 2*: Internet use behavior is positively correlated with prohibitive behavior among adolescents.

## 2 Method

### 2.1 Participants

Data for this study came from the China Education Panel Survey (CEPS). The data from the baseline survey and the monitored data for the second round were conducted in the academic years of 2013–2014 and 2014–2015, respectively. When the missing ratio is less than per cent, the difference is likely to be small by any missing data treatments (Liu, 2019). Therefore, this study was less than 10%, and the missing samples were deleted, so the data had strong credibility. The first survey (T1) was conducted among students in grade 7 and grade 9. The follow-up data were collected from 9,449 samples of the second test time (T2). There were 317 missing values between the two waves (part of it); they were eliminated since it was not possible to pair the two-stage samples individually. After removing this, 9,132 samples remained.

### 2.2 Measures

#### 2.2.1 Internet use behavior

Drawing on Kabasakal’s (2015) research approach, the study uses time spent on problematic Internet use as a predictor, analyzing problematic Internet use behavior among students based on variables such as smoking, drinking, gambling behavior, Internet use duration, daily time spent on the Internet, and Internet use for academic purposes. Daily time spent on the Internet has a strong impact on the Internet use dimension. Therefore, this study selected junior high school students’ daily Internet usage duration to measure their Internet use behavior. Internet use behavior from the student questionnaire: surfing the Internet and playing games, from Monday to Friday, from no to more than 4 h a day, is divided into a 6 -point scale (1. No; 2. less than 1 h; 3. about 1–2 h; 4. about 2–3 h; 5. about 3–4 h; 6. about 4 h). Surfing the Internet and playing games on weekends were scored from none to more than 8 h per day, corresponding to codes of 1–6 (1. No; 2. less than 2 h; 3. about 2–4 h; 4. about 4–6 h; 5. about 6–8 h; 6. about 8 h or more).

#### 2.2.2 Adolescent behavioral development

Promotive and prohibitive behaviors for adolescent behavioral development were obtained from the baseline and follow-up data. The proxy indicators of promotive behavior included three items: helping the elderly to do things, observing public order, being sincere and friendly to others. Specifically, the indicators are (1) “helping the elderly with tasks,” which reflects promotive efforts in online scenarios; (2) “observing public order,” representing adherence to communal norms that motivates collective progress and overcomes societal barriers; and (3) “being sincere and friendly to others,” embodying authentic interpersonal engagement that encourages mutual goal pursuit in virtual interactions. Prohibitive behaviors were assessed by questions (e.g., “Have you had any of the following behaviors in the past year?”). Adolescent behavioral development was measured by “positive and negative behaviors performed in the past year” on a 5-point scale ranging from never to always (Wu and Zhang, 2018).

#### 2.2.3 Data analysis

To mitigate the bias caused by the characteristics and methods of the data analysis, the single-factor method was used to test the data gathered. It is generally believed that a single factor cannot explain more than 40% of the variation (Tang and Wen, 2020). The two measurements’ respective findings were 34.59 and 38.24%, which were below 40%. The variability at time points below the critical criterion of 40% was 34.59 and 38.24%, respectively, which was explained by the first factor of the results of the T1 and T2 measurements. This strongly suggests that the data in this study showed no evident common methodological deviations. Therefore, there was no evident common method bias in the results of both surveys (Xie et al., 2021). At the same time, the VIF of each variable is less than 10, indicating that there is no serious multicollinearity.

## 3 Results

### 3.1 Descriptive statistics

The results of Pearson product–moment correlation analysis showed that Internet use behavior at the two-time points (T1, T2) was significantly correlated with each dimension of adolescent behavior development (promotive behavior and prohibitive behavior). Table 1 shows that Internet use behavior can stably and continuously influence adolescent behavioral development. T1 and T2 show that promotive behaviors were negatively correlated with Internet use behaviors, indicating that Internet use behaviors had a certain restrictive effect on the development of positive behaviors in adolescents. Prohibitive behaviors were positively correlated with Internet use behaviors, indicating that Internet use behavior had a certain promoting effect on the development of adolescent negative behaviors in T1 and T2. We can observe a close relationship between Internet use behavior and the development of adolescent behavior.

**Table 1:** Correlation matrix between Internet use behavior and adolescent behavioral development.

	*M*	SD	1	2	3	4	5
1. Promotive behavior (T1)	2.48	0.51					
2. Prohibitive behavior (T1)	1.10	0.25	−0.21***				
3. Promotive behavior (T2)	2.44	0.53	0.40***	−0.19***			
4. Prohibitive behavior (T2)	1.17	0.33	−0.19***	0.31***	−0.25***		
5. Internet use behavior (T1)	1.82	0.97	−0.12***	0.17***	−0.12***	0.17***	
6. Internet use behavior (T2)	2.42	1.23	−0.11***	0.19***	−0.17***	0.23***	0.42***

T1 represents the baseline data, T2 represents the second round data; *p < 0.05, **p < 0.01, ***p < 0.001, the same below.

The average score for promotive behavior in T1 and T2 is 2.48 and 2.44, respectively, and the standard difference is 0.51 and 0.53. This indicates that at time points T1 and T2, junior high school students’ performance in promoting behavior is basically average, and the standard deviation is small, while the average score of prohibitive behaviors is lower at T1 or T2. This indicates a positive overall behavioral development in junior high schools, yet there is still room for further improvement. Since the main objective of this study is to understand the general mechanisms for the development of Internet use behavior and adolescent individual behavior, control variables are not taken into account.

### 3.2 Cross-lagged regression analysis

#### 3.2.1 Cross-lagged regression analysis of Internet use and adolescent promotive behaviors

**Figure 1:**
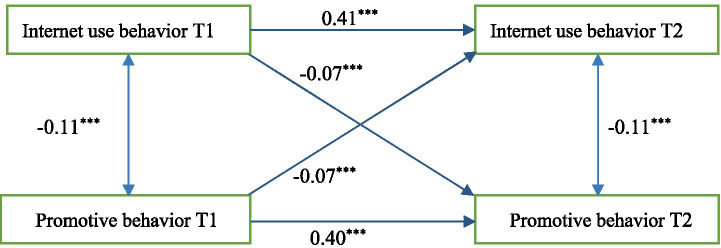
Cross-lagged regression model plot of Internet use behavior and adolescent promotive behavior.

Based on the previous correlation analysis, the cross-lagged model of Internet use behavior and adolescent behavior development was established to clarify the longitudinal relationship between Internet use behavior and adolescent behavior development and to explore the influencing mechanism. The results of the cross-lagged model between Internet use behavior and adolescent promotive behavior found that the prediction coefficient of T1 Internet use behavior on T2 promotive behavior was very significant (see Figure 1), with a cross-time negative predictive effect (*β* = −0.07, *p* < 0.001). The predictive coefficient of promotive behavior at T1 to Internet use behavior at T2 was very significant, with a negative predictive effect across time points (*β* = −0.07, *p* < 0.001). Results showed that there was a significant interaction between Internet use behavior and adolescent promotive behavior.

#### 3.2.2 Cross-lagged regression analysis of Internet use and prohibitive behaviors among adolescents

**Figure 2:**
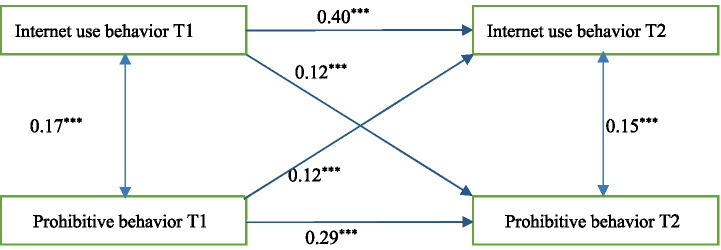
Cross-lagged model plots of Internet use behavior and adolescent prohibitive behavior.

The results of the cross-lagged model between Internet use behavior and prohibitive behavior found that the prediction coefficient of T1 Internet use behavior on T2 prohibitive behavior was very significant (see Figure 2), with a cross-time positive predictive effect (*β* = 0.12, *p* < 0.001). Prohibitive behavior at T1 had a significant effect on predicting Internet use behavior at T2, with a positive effect across time points (*β* = 0.12, *p* < 0.001). Results showed that there was a significant interaction between Internet use behavior and prohibitive behaviors among adolescents.

In this study, 50% of the subjects were randomly selected from the research samples to conduct a sensitivity test, which is similar to the results of the whole sample, so the results of this study are relatively robust.

## 4 Discussion

To explore the relationship between Internet use behavior and adolescent behavior development by constructing a cross-lagged model, the study found a high correlation between Internet use behavior and adolescent behavioral development.

First, Internet use behavior induces a negative interaction procedure: Internet use behavior at T1 reduces T2 promotive behaviors while increasing T2 prohibitive behaviors. Second, adolescent behaviors were inversely associated with Internet use behaviors: promotive behaviors at T1 reduced Internet use behaviors at T2; Prohibitive behaviors at T1 increased Internet use behaviors at T2. Third, interaction exists between Internet use behavior and adolescent behavioral development. The results show that Internet use behavior is closely related to the development of adolescent behavior, and there is a complex relationship between effect and reaction. The beautiful vision of the Internet promoting student development can be realized by observing the relationship between “Internet+” and student development through the lenses of system theory and complexity theory.

Chaos theory is the science that studies nonlinear dynamical systems, revealing that many phenomena that appear random and unpredictable are in fact governed by deterministic laws. Lorenz introduced the “butterfly effect” and popularized the concept of deterministic chaos. Modern chaos theory amplifies tiny changes in chaotic systems’ initial conditions over time, resulting in markedly different outcomes. Deterministic rules govern the evolution of chaotic systems, but they cannot predict long-term behavior. Moreover, chaos arises from its underlying deterministic nature, which includes attractors. Put differently, the different patterns generated by small variations in initial conditions are drawn toward specific regions known as chaotic or strange attractors. These attractors define the boundaries of the chaotic dynamics of a given system (Froidure et al., 2020). According to chaos theory in systems science, there is a complex, dynamic, interactive relationship between adolescents’ Internet use behaviors and their behavioral development. The process of adolescent behavioral development can be regarded as a complex dynamic system. The results of the cross-lagged regression analysis in this study, particularly the significant predictive effects of T1 Internet use behavior on T2 promotive and prohibitive behaviors, embody the “sensitivity to initial conditions” or “butterfly effect” in chaos theory. This implies that subtle differences in adolescents’ early Internet use behavior may be amplified during subsequent developmental processes, leading to significant differentiation in their behavioral patterns and ultimately directing them toward divergent developmental trajectories. The coexistence of these two behavioral patterns suggests that the adolescent behavioral development system is in such a critical state. It is neither static nor chaotic but rather harbors opportunities for change within stability. At the individual level, however, it may lead to starkly different directions due to a minor initial difference or a random event (such as a successful intervention or a major setback).

### 4.1 The behaviors caused by Internet use behaviors

By constructing a cross-study model, we analyzed the negative impact of Internet use behavior on adolescent behavior development. Internet use behavior at points-in-time T1 negatively predicted promotive behavior at points-in-time T2 and positively predicted prohibitive behavior at T2. The longer the time spent using the Internet, the more unfavorable the behavior development of adolescents. In addition, the self-control ability of adolescents is not strong enough to regulate their network behavior, which can easily to lead to deviation from behavior development. This research result is consistent with the results of Ning et al., who concluded that the longer the peer Internet use time, the higher the probability of adolescent Internet addiction (Ning et al., 2021).

More Internet use leads to fewer interactions with family members, smaller social circles, and increased depression and loneliness (Kraut et al., 1998). Teenagers are curious about the Internet and tend to experience new things, often attracted by online games, competitive activity programs, and virtual spaces. Competitive confrontation and even violent acts in games influence teenagers, drawing them from virtual space into real life and forming prohibitive behaviors. Simultaneously, the ability of adolescents to exercise self-control is relatively weak. Facing the temptation brought by the information environment or the induction of peers, they often plunge into it without thinking. When they enter the virtual space, they complete self-transcendence in the interaction with the subjects in the virtual field. This transcendence produces behavioral deviation in the real-life world. There may be a decline in real-world interactions.

### 4.2 Adolescent behavior is significantly related to Internet use

Through the cross-lagged model analysis, the Internet use behavior of T1 has a very significant effect on both promotive and prohibitive behaviors. Conversely, adolescents’ behavior orientation also influenced Internet use behaviors. The promotive behaviors reduced the time and frequency of Internet use, while the prohibitive behaviors increased the time and frequency of Internet use. This phenomenon is specifically related to the ability of adolescents to shift their behavioral model. Edmund Husserl believed that from the initial recognition of the individual to the appearance of the behavior, it needs to go through four levels of perceptual behavior, visual behavior, image behavior, and object behavior; the action result is the representation of the object behavior (Xiong and Su, 2022).

Perceptual behavior, visual behavior, and image behavior determine the performance and presentation results of object behavior. If the purpose and usefulness of the perceived behavior deviate, the intuitive behavior and the apparent behavior that enter the individual’s field of vision also deviate from the basic norms of behavior, which leads to the obstructed transformation of behavior and the emergence of prohibitive objectivized behavior. This is mainly because the ability of adolescents to judge behavior needs to be further developed, and intuitive behavior and apparent behavior are often based on the will of self-feelings and may not desire or want to produce behavioral results on the subjective will. For example, youth find it difficult to solve problems in their study life and think that the Internet can provide the perfect solutions, when they accidentally come into contact with one kind of entertainment game during the process of using the Internet, they might forget the original intention and the gameplay self-realization, leading to cognitive behavioral transformation to the objective behavior of a series of mistakes.

Therefore, schools and families should provide proper guidance on Internet use behavior during adolescent development, helping them to realize completion from perception behavior, intuitive behavior, effective transformation to objective behaviors, fostering positive interactions between adolescent behavior and Internet use.

### 4.3 The interaction between Internet use and adolescent behavioral development

The results indicated that there was a significant interaction between Internet use behavior, promotive behavior, and prohibitive behavior. The Internet influences individual behavior, and the virtual world of the Internet influences individual behavior as well. There may be a two-way, timely feedback interaction between Internet use behavior and individual behavior. Students talk to their teachers, express their desires, and form relationships. The dialogue of the communication subject’s behavior is to find the channel to satisfy oneself, to achieve comfort. Goulart and Kafure’s (2019) research reveals that young people spend considerable time on the internet and are profoundly shaped by their living environment, especially the influence of peers. Adults tend to hold a predominantly negative perception regarding some adolescents’ behaviors, a perception that is not substantiated when compared to actual youth practices (Goulart and Kafure, 2019).

However, teenagers use the Internet to realize the sense of self-satisfaction in the way of satisfying self-requirements, which will lead to strengthening the persistence of behavior. The environment plays a decisive role in forming stable behavior. The Internet, with its fantasy and extremely attractive virtual space, is extremely attractive to teenagers.

### 4.4 Limitations and future directions

Cross-lagged studies on the relationship between Internet use behavior and adolescent behavior development have yielded interaction findings. However, there are still some shortcomings as follows: First, the variables are affected by systematic errors (Lu et al., 2018). In future research, we should combine the methods of field investigation, site inspection, and multi-subject reports to further verify the research results. Second, only two follow-up datasets were used. Finally, Internet use behavior and adolescent behavior development are complex and multi-factor systems composed of multiple elements. Relevant control variables should be considered in future research to better understand the mutual mechanism between Internet use behavior and adolescent development in a comprehensive, dynamic, and continuous manner. Although the researchers focus on general mechanisms, background influences may exist. Future work could add controls to refine understanding of specific pathways and subgroups.

### 4.5 Research significance

This study has significant research value since it sheds light on adolescents’ behavior patterns in the online environment and offers a scientific foundation for healthy Internet use. Furthermore, by preventing Internet addiction and other negative behaviors, this study may assist parents and educators in better guiding teenagers’ use of the Internet in a scientific manner and so promoting their physical and mental well-being.

## 5 Conclusion

Based on the above findings, we found that Internet use behavior affects adolescent promotive behaviors as well as prohibitive behaviors. Similarly, adolescent behavior also influences Internet use behavior. These findings indicate that teenagers must master the necessary Internet technology in the digital age, as this mastery not only serves as essential literacy for their development but also helps transform their Internet use behaviors to align with the evolving needs of digital technology. At the same time, adolescents should be correctly guided to use the Internet rationally to provide quality resources and services to solve problems in studies and life.

## Data Availability

The raw data supporting the conclusions of this article will be made available by the authors, without undue reservation.
